# Associations among leadership, resources, and nurses’ work engagement: findings from the fifth korean Working Conditions Survey

**DOI:** 10.1186/s12912-023-01331-8

**Published:** 2023-06-05

**Authors:** Eunkyung Kim, Ji Yea Lee, Seung Eun Lee

**Affiliations:** 1 College of Nursing, Eulji University Uijeongbu Campus, 712, Dongil-ro, 11759 Uijeongbu-si, Gyeonggi-do South Korea; 2 College of Nursing, Yonsei University, 50-1 Yonsei-ro, Seodaemun-gu, 03722 Seoul, South Korea; 3 Mo-Im KIM Nursing Research Institute, College of Nursing, Yonsei University, 50-1 Yonsei-ro, Seodaemun-gu, 03722 Seoul, South Korea

**Keywords:** Nurses, Leadership, Resource, Work environment, Work engagement

## Abstract

**Background:**

Nurses’ work engagement has received extensive attention due to its positive impacts on individual and organizational outcomes, including patient safety and quality care in healthcare organizations. Although nurse managers’ leadership and a variety of resources have been identified as important factors of nurses’ work engagement, these relationships have not been well understood in Korean nursing contexts. The purpose of this study was to examine the associations among nurse managers’ leadership, resources, and work engagement among Korean nurses after controlling for nurses’ demographic and work-related characteristics.

**Methods:**

This is a cross-sectional study using data from the fifth Korean Working Conditions Survey. Using a sample of 477 registered nurses, we employed hierarchical linear regression analyses. Nurse managers’ leadership, job resources (organizational justice and support from peers), professional resources (employee involvement), and personal resources (meaning of work) were examined as potential predictors of nurses’ work engagement.

**Results:**

We found that nurse managers’ leadership (β = 0.26, 95% confidence interval [CI] = 0.17–0.41) was the strongest predictor of nurses’ work engagement, followed by meaning of work (β = 0.20, 95% CI = 0.07–0.18), organizational justice (β = 0.19, 95% CI = 0.10–0.32), and support from peers (β = 0.14, 95% CI = 0.04–0.23). Employee involvement was not a statistically significant predictor of nurses’ work engagement (β = -0.07, 95% CI = -0.11–0.01).

**Conclusions:**

Our findings suggest that comprehensive approaches are required to promote nurses’ work engagement. Considering that nurse managers’ leadership was the strongest predictor of nurses’ work engagement, nurse managers should demonstrate supportive leadership behaviors such as acknowledging and praising their unit nurses’ work performance. Furthermore, both individual- and organizational-level strategies are necessary for nurses to be engaged at work.

## Introduction

The importance of nurses’ work engagement has been emphasized in the nursing literature [1]. Work engagement refers to one’s psychological state about work, which is characterized by vigor, dedication, and absorption [2]. Work engagement indicates employees’ status of being passionate about and immersed in their work. Previous research has shown that nurses’ work engagement is associated with better individual and organizational outcomes, such as improved job satisfaction and job performance and lower turnover intention [3–6]. Researchers have found that highly engaged nurses were more likely to be competent in job-related tasks and to behave in ways that help achieve organizational goals [6]. Furthermore, previous research has shown that nurses’ work engagement plays an important role in better patient safety and quality care [6, 7]. For example, highly engaged nurses tended to discuss strategies to prevent medical errors and to speak up when they had patient safety concerns [8]. Due to the importance of nurses’ work engagement, researchers have identified its related factors [3, 4] and shown that nurse managers’ leadership and various resources are closely associated with work engagement. However, the comprehensive relationships among nurse managers’ leadership, resources, and nurses’ work engagement are not yet fully understood.

Managers’ supportive leadership, defined as behaviors that satisfy employees’ needs [9], is known to be an important factor for employees’ work engagement because such leadership helps employees trust that the workplace is safe to become engaged in their work [10]. Empirical evidence from non-healthcare fields has demonstrated that Chinese employees in a trading company were more likely to be engaged in their work when they perceived supervisors as having a supportive leadership style [11]. Similar findings were revealed for employees from various fields in Austria, Germany, and Switzerland in a study that used European Working Conditions Survey data [12]. Considering that supportive leadership has gained great attention from various research fields [9] and demonstrated a positive relationship with work engagement [11, 12], it seems possible that this relationship exists in the nursing context as well. Therefore, research that investigates the relationships between nurse managers’ supportive leadership and nurses’ work engagement is needed.

**Fig. 1:**
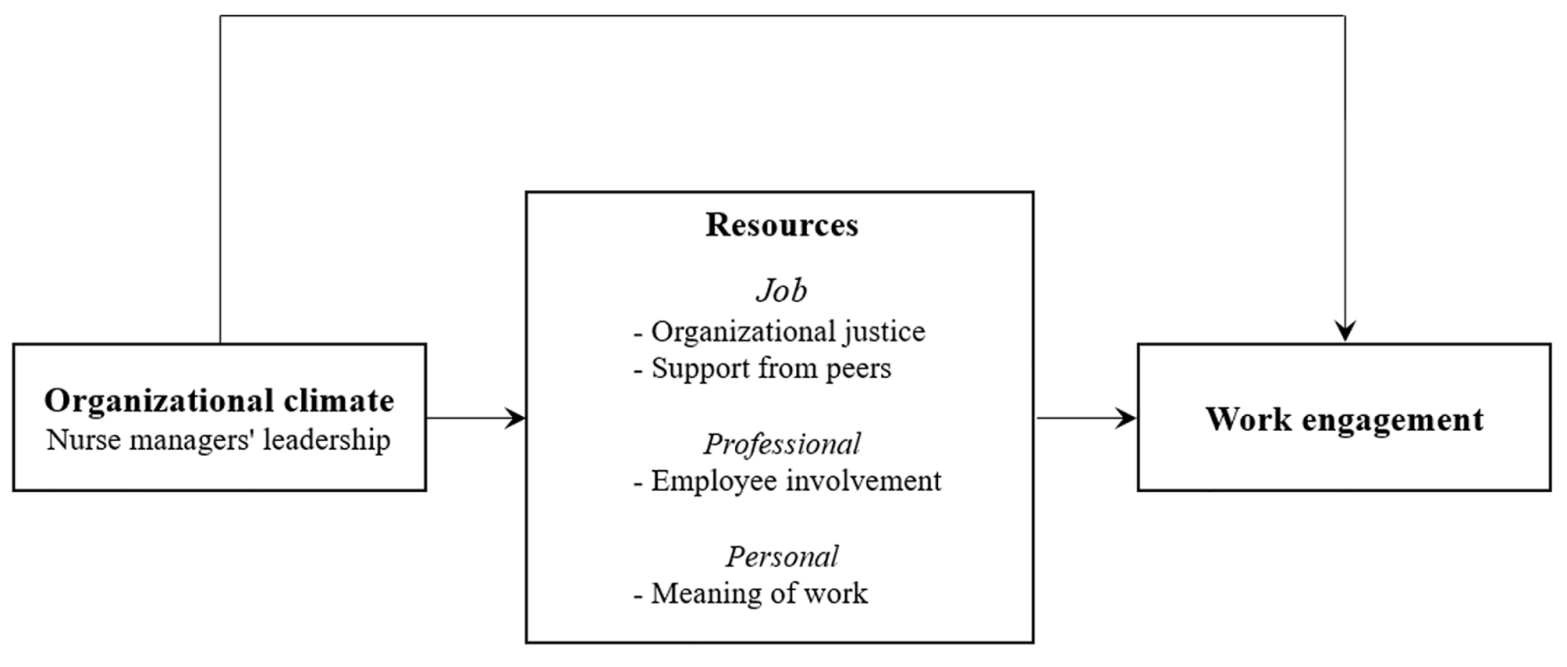
Conceptual framework

A recent systematic review suggested the Nursing Job Demands-Resources (NJD-R) model [4] to demonstrate the associated factors and consequences of work engagement in nursing practice. This model postulates that unit managers’ leadership affects nurses’ work engagement, and this relationship is influenced by various resources (i.e., job, professional, and personal resources) [4]. Guided by this model and the literature, we set the following hypothesis for this study: nurse managers’ leadership and various resources are associated with nurses’ work engagement. As shown in Fig. 1, we develop a conceptual framework to investigate how resources are related to the associations between nurse managers’ leadership and nurses’ work engagement.

Job resources focus on organizational or social aspects of jobs, such as organizational justice and support from peers [13]. Organizational justice refers to employees’ perceptions of the fairness of management’s decisions and actions [14], and a positive perception of organizational justice makes employees more immersed in their work [15]. There is evidence showing that organizational justice significantly impacts nurses’ work engagement [5, 16, 17]. Support from peers, another type of job resource, enhances nurses’ work engagement by helping them achieve work-related goals [18]. Previous studies found peer support to be associated with the work engagement of Portuguese [19] and Chinese [20] nurses.

Professional resources refer to resources that support nurses in practicing according to their professional standards and achieving their goals as healthcare providers [4], and these resources provoke motivation for work engagement [13]. Employee involvement is a professional resource that indicates employees’ participation in decision-making in their workplace [21]. It inspires in employees a sense of responsibility for work, in turn motivating them to be engaged in their work [22], and significant relationships between employee involvement and nurses’ work engagement were revealed in studies in China [23] and Belgium [24].

Personal resources indicate psychological traits that help one control one’s environment successfully [25]. Personal resources are internal factors that are oriented around employees themselves [25]. Meaning of work is one example of a personal resource and refers to finding existential meaning in one’s work experience [26]. Such meaning encourages employees to be more engaged in their work by helping them establish personal identity in the work context [27]. Researchers have found significant correlations between meaning of work and employees’ work engagement in studies in Spain [28] and the US [29].

Despite the aforementioned studies on various factors influencing nurses’ work engagement, there is a lack of research that comprehensively examines the effect of nurse managers’ leadership and various types of resources on nurses’ work engagement. Thus, this study aimed to investigate the relationships among nurse managers’ leadership, resources, and work engagement among Korean nurses. Our study might provide a better understanding of the complex phenomenon related to work engagement within nursing practice and insights for enhancing nurses’ work engagement.

## Methods

### Design

This cross-sectional, correlational study was conducted using publicly available, anonymized data from the fifth Korean Working Conditions Survey (KWCS) [30].

### Description of primary data

The fifth KWCS was benchmarked according to the European Working Conditions Survey and the Labor Force Survey, and its quality is controlled by the Occupational Safety and Health Research Institute, a government agency of South Korea [31]. The purpose of the KWCS was to identify working conditions affecting health and safety among Korean workers who were economically active employees aged 15 years or older. Study samples were selected through a stratified sampling method based on data from the Population and Housing Census [32]. For data collection, trained interviewers visited study participants and conducted interviews, and the participants were informed about the voluntary nature of their participation in the study and the confidentiality of their responses. The fifth KWCS data collection was performed in 2017 in 8 cities and 9 provinces in South Korea [31]. The survey consisted of questions about labor intensity, stress, working patterns, emotional labor, education and training, job satisfaction, health problems, and exposure to risk factors. A total of 50,205 South Koreans participated in the survey.

**Fig. 2:**
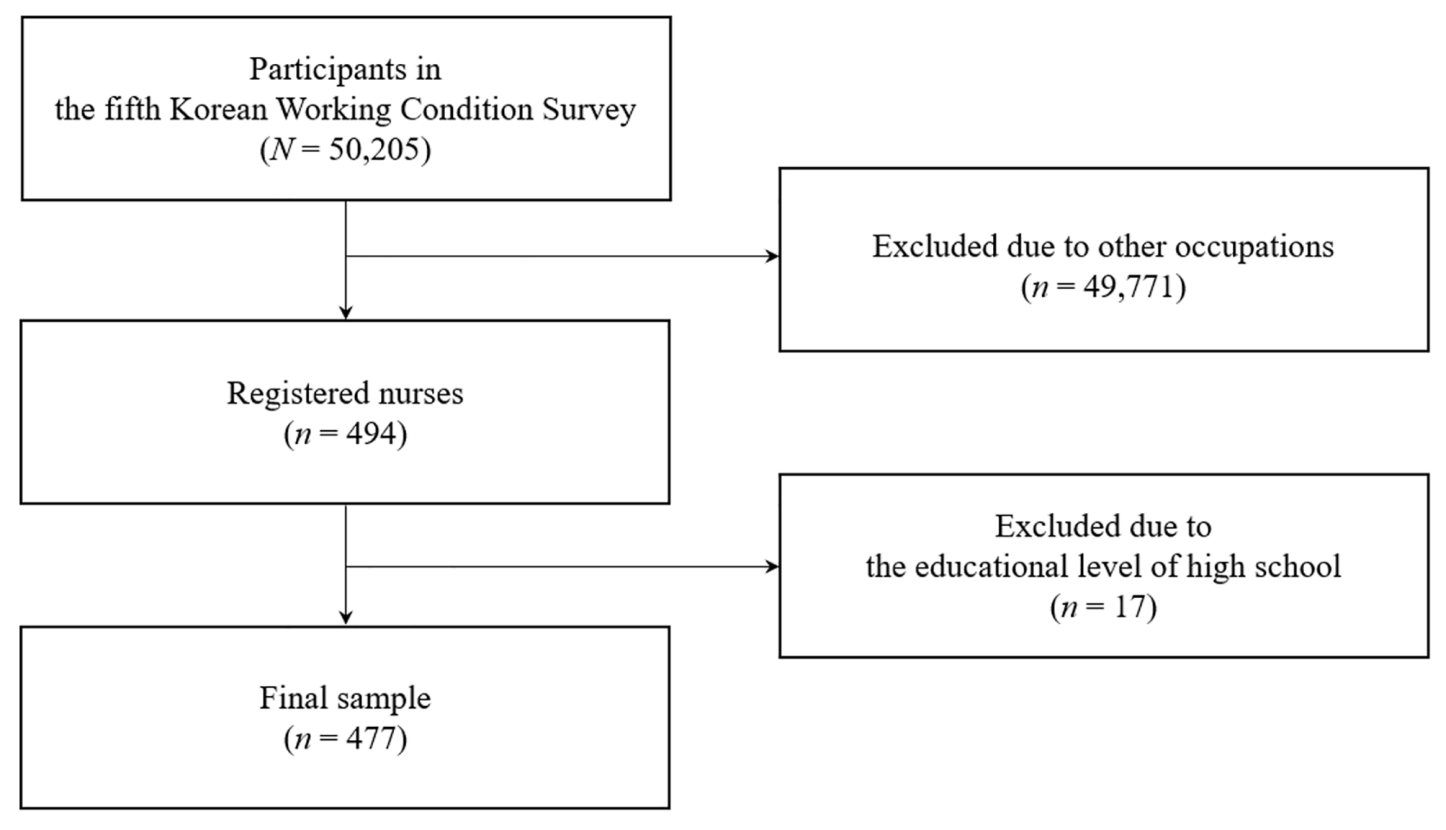
Flowchart of study sample

The inclusion criterion for the current study was registered nurses (RNs) with an associate degree or higher. In the original data set, 494 respondents indicated that they were RNs. However, we found that 17 of them reported their highest educational degree as a high school diploma, which is not possible in South Korea because RNs must have at least an associate’s degree or higher by the law [33]. Thus, we excluded data from the 17 respondents, leaving a final sample of 477 RNs for this study (Fig. 2).

### Measures

In the original survey, all items were measured on a 5-point Likert scale (1 = always or strongly agree, 5 = never or strongly disagree). For statistical analyses, negatively worded items were reverse-coded.

Work engagement was measured using three items from the third version of the Copenhagen psychosocial questionnaire (COPSOQ III) [34]. The items were as follows: (a) At my work, I feel bursting with energy; (b) I am enthusiastic about my job; and (c) I am immersed in my work. For this study, the Cronbach’s alpha value was 0.85.

Guided by previous research [12], nurse managers’ leadership was measured using the following six items: (a) Your immediate boss respects you as a person; (b) Your immediate boss gives you praise and recognition when you do a good job; (c) Your immediate boss is successful in getting people to work together; (d) Your immediate boss is helpful in getting the job done; (e) Your immediate boss provides useful feedback on your work; and (f) Your immediate boss encourages and supports your development. For this study, the Cronbach’s alpha value was 0.89.

Organizational justice was measured using three items from the COPSOQ III [34]. The items were (a) Employees are appreciated when they have done a good job; (b) Conflicts are resolved in a fair way; and (c) The work is distributed fairly. The Cronbach’s alpha value was 0.77 for this study.

Support from peers was measured using a single item from the COPSOQ III [34]. The item was “Your colleagues help and support you,” which has previously been used to measure peer support in a Korean population [35].

Meaning of work was measured using a single item from the COPSOQ III [34]. The item was “You have the feeling of doing meaningful work,” which has been used to assess the meaning of work in previous research [36].

Guided by previous literature [37, 38], employee involvement was assessed using the following four items: (a) You are consulted before targets for your work are set; (b) You are involved in improving the work organization or work processes of your department or organization; (c) You can influence decisions that are important for your work; and (d) You are able to apply your own ideas in your work. Cronbach’s alpha value was 0.87 for this study.

Sample characteristics included nurses’ gender (male or female), age, educational level (associate, or bachelor’s or higher degree), and job tenure (the length of time at the current workplace). Work-related characteristics consisted of workplace location, workplace size, working hours per week, shift work, and salary. The location of the workplace was dichotomized into metropolitan (capital and 7 cities), and non-metropolitan areas. The original KWCS data set showed that workplace sizes fell into three categories based on the number of employees: small (1–9), medium (10–249), and large (≥ 250), and these categories were used for analysis in the current study. Working hours per week were measured in units of hours, which were dichotomized into ≤ 40 and ≥ 41 for data analysis because weekly working hours cannot exceed 40 h under the Korean Labor Standards Act [39]. Respondents who answered “yes” to the question “I am a shift worker” were categorized as shift workers, and those who answered “no” to the question were categorized as non-shift workers. Salary was measured as income in recent months in units of 10,000 Korean won. Age, job tenure, and salary were continuous variables.

### Data analysis

Descriptive statistics were used to describe the sample and work-related characteristics. Pearson’s correlation analysis was conducted to examine the relationships between key study variables. Finally, hierarchical linear regression analyses were carried out to examine the associations among nurse managers’ leadership, various resources, and nurses’ work engagement. In the first step, the association between nurse managers’ leadership and work engagement was tested (Model 1). In the second step, resources were added (Model 2) into Model 1 to examine the effect of each resource on work engagement after accounting for the effect of nurse managers’ leadership. We controlled for age [23, 40], workplace location [41, 42], working hours per week [43], shiftwork [41], and salary [44] since these variables were reported to be related to nurses’ work engagement. All analyses were performed using SPSS version 26.0 with *p* < 0.05 indicating statistical significance.

### Ethics considerations

The Institutional Review Board of the relevant university determined exempt review for this study (#xxx blinded for review).

## Results

### Sample and work-related characteristics of participants

As shown in Table 1, most participants were female (98.3%), with an average job tenure of 6.82 years (SD = 6.25). The mean age of the participants was 37.21 years old (SD = 9.02), which is similar to the South Korean nursing population [45]. Approximately 60% of participants had a bachelor’s degree or higher. Less than half (43.8%) worked in medium-sized workplaces, and approximately 53% worked in non-metropolitan areas. More than half (54.5%) worked 40 h or less per week. Approximately one-third (35.6%) of the participants were shift workers, and the mean salary of all participants was 252.01 10,000 Korean won (SD = 74.35).

**Table 1:** Sample and Work-related Characteristics of Participants (N = 477)

Characteristics	Categories	M ± SD or *n* (%)
Sample characteristics		
Gender	Female	469 (98.3)
	Male	8 (1.7)
Educational level	Associate	193 (40.5)
	Bachelor’s or higher	284 (59.5)
Age (year)	–	37.21 ± 9.02
Job tenure (year)	–	6.82 ± 6.25
Work-related characteristics		
Workplace location	Metropolitan	223 (46.8)
	Non-metropolitan	254 (53.2)
Workplace size ^a^	Small (1–9)	186 (39.0)
	Medium (10–249)	209 (43.8)
	Large (≥ 250)	81 (17.0)
Working hours per week	≤ 40	260 (54.5)
	≥ 41	217 (45.6)
Shift work	Yes	170 (35.6)
	No	307 (64.4)
Salary (10,000 Korean won)	–	252.01 ± 74.35

*M* mean, *SD* standard deviation

^a^ Frequency of missing values was 1, workplace size was classified based on the number of employees

### Descriptive statistics and correlations between key study variables

Table 2 displays the descriptive statistics of and correlation coefficients between the key study variables. The mean score of support from peers was the highest (M = 3.90, SD = 0.64) and that of employee involvement was the lowest (M = 3.14, SD = 0.82). Bivariate correlation analyses showed that except for employee involvement (*r* = 0.06, *p* = 0.20), all other variables were significantly correlated with work engagement, ranging from 0.11 to 0.56 (*p* < 0.05). Additionally, we found no significant correlations between age and work engagement (*r* = -0.02, *p* = 0.72).

**Table 2:** Correlations and Descriptive Statistics for Key Study Variables (N = 477)

Variables	1	2	3	4	5	6
Nurse managers’ leadership	–	–	–	–	–	–
Organizational justice	0.56^**^	–	–	–	–	–
Support from peers	0.46^**^	0.38^**^	–	–	–	–
Employee involvement	0.15^*^	0.22^**^	0.17^**^	–	–	–
Meaning of work	0.27^**^	0.23^**^	0.36^**^	0.11^*^	–	–
Work engagement	0.46^**^	0.40^**^	0.38^**^	0.06	0.34^**^	–
M	3.77	3.68	3.90	3.14	3.66	3.68
SD	0.52	0.53	0.64	0.82	0.96	0.59

*M* mean, *SD* standard deviation

^*^p < 0.05; ^**^p < 0.001

### Hierarchical linear regression analyses

Table 3 shows the results of hierarchical linear regression analyses. In Model 1, nurse managers’ leadership was significantly associated with nurses’ work engagement (β = 0.48, 95% confidence interval [CI] = 0.44–0.63), and this model explained 24% of the variance in nurses’ work engagement. The results indicate that when nurses perceived their nurse managers’ leadership as being supportive, they were more likely to be engaged in their work. After introducing resource variables in Model 2, nurse managers’ leadership remained the strongest predictor of nurses’ work engagement (β = 0.26, 95% CI = 0.17–0.41). By examining the R^2^ change, we found that resources explained an additional 9% of the variance in nurses’ work engagement. Among resources, meaning of work (β = 0.20, 95% CI = 0.07–0.18), organizational justice (β = 0.19, 95% CI = 0.10–0.32), and support from peers (β = 0.14, 95% CI = 0.04–0.23) were positively related to the work engagement of nurses; however, employee involvement was a non-significant predictor of nurses’ work engagement (β = -0.07, 95% CI = -0.11–0.01).

**Table 3:** Hierarchical linear regression analyses for work engagement (N = 477)

Variables	Model 1		Model 2	
	β	95% CI	β	95% CI
Age	-0.04	-0.01–0.00	-0.01	-0.01–0.01
Workplace location^†^	-0.09^*^	-0.21 – -0.01	-0.08^*^	-0.20 – -0.00
Working hours per week^‡^	-0.05	-0.17–0.05	-0.05	-0.16–0.04
Shift work^§^	0.00	-0.18–0.12	-0.00	-0.11–0.11
Salary	-0.05	-0.00–0.00	-0.08	-0.00–0.00
Nursing managers’ leadership	0.48^**^	0.44–0.63	0.26^**^	0.17–0.41
Organizational justice			0.19^**^	0.10–0.32
Support from peers			0.14^*^	0.04–0.23
Employee involvement			-0.07	-0.11–0.01
Meaning of work			0.20^**^	0.07–0.18
Change in R^2^	0.24^**^		0.09^**^	
R^2^	0.24^**^		0.33^**^	

*β* standardized coefficient, *CI* confidence interval

^†^ Reference: Metropolitan; ^‡^ Reference: ≤ 40; ^§^ Reference: No

^*^p < 0.05; ^**^p < 0.001

## Discussion

This study examined the relationship among nurse managers’ leadership, various resources, and nurses’ work engagement after controlling for nurses’ demographic and work-related characteristics. We found that nurse managers’ leadership, meaning of work, organizational justice, and support from peers were significantly related to work engagement among South Korean nurses.

Similar to the findings from a previous study [11], we found that nurse managers’ leadership was a significant predictor of nurses’ work engagement. Several studies have demonstrated that nurse managers’ leadership could play a key role in encouraging nurses’ work engagement [3] through various pathways, such as optimizing working conditions, satisfying their psychological needs, or being a role model [46]. The measure used to assess leadership in this study focused on expressing interests in their staff and taking into account their needs in the workplace [9]. Such leadership can meet nurses’ preferences and psychological needs [46] and foster a friendly work environment [47]. This, in turn, can enhance nurses’ passion and immersion in their work, in other words, their work engagement. Thus, nurse managers should focus on promoting nurses’ well-being and creating a positive workplace climate with respect, recognition, and support among nurses to enhance nurses’ work engagement.

Consistent with findings from previous research [48], we found that meaning of work was a significant predictor of nurses’ work engagement, indicating that nurses were more likely to be engaged in their work when they perceived their work to be meaningful, valuable, and worthwhile. The meaningfulness of work arises when people fully understand their work purpose [49], and it is closely associated with their values, motivations, and beliefs toward the work [50]. Individual nurses should find their desires through work and the significance of work in their lives to improve work engagement. Nurse managers can help nurses link the meaningfulness of their work, such as a sense of fulfillment or having a professional specialty, to their values and motivation. Furthermore, organizations need to understand the importance of meaning of work in nurses’ work engagement and provide better working conditions wherein nurses can find meaningfulness in their work, which will, in turn, improve their work engagement.

We found that nurses who perceived their organizations as treating them fairly were more engaged in their work. This finding is consistent with previous literature showing a positive association between organizational justice and work engagement among Chinese nurses [5, 17]. Organizational justice can contribute to employees’ positive attitudes toward work [17]. Nurses perceive organizational justice in the workplace when problem-solving processes or compensation criteria are explicitly shared with them [17]. Therefore, organizational justice can be achieved by establishing a work environment with a fair and equitable reward system, shared guidelines or policy for decision-making, and clear processes for problem-solving [51], which could lead to nurses’ higher work engagement.

Additionally, consistent with findings from previous research in various countries [19, 20, 52], we found that support from peers was positively associated with nurses’ work engagement. The positive relationship might be due to the various roles of peers in the workplace. For instance, peers can provide emotional support by being one’s companions or provide tangible support, such as alleviating others’ workloads [53]. Hence, it should be encouraged to create a workplace culture where mutual support among nurses can be facilitated. Educational interventions regarding teamwork and collaboration can be developed as a strategy for creating a supportive work atmosphere [54].

In contrast to our expectations, in the present study, employee involvement was not a significant predictor of nurses’ work engagement. This might be because South Korean nurses might feel burdened by being involved in decision-making or suggesting ideas due to heavy workloads [55]. For example, the nurse-to-patient ratio, which is an indicator of nurses’ workload [56], is, on average, 16.3 in South Korea, whereas it is 5.3 in the US and 7.9 in Switzerland [55]. The excessive workload may make it difficult for nurses to complete even assigned tasks under time constraints [55]. Interestingly, the relationships between employee involvement and work engagement were inconsistent with previous studies. For example, one US study reported an insignificant relationship between employee involvement and nurses’ work engagement [57], while in a Belgian study, employee involvement was significantly and positively related to nurses’ work engagement [24]. As the empirical literature has reported conflicting results, further studies are needed to better understand the relationship between employee involvement and the work engagement of nurses.

### Limitations

Several limitations should be noted. First, this is a cross-sectional study, meaning that it cannot infer causality among the variables. Second, although we used data from samples from a national database, the generalizability of this study’s results might be limited due to the low proportion of male nurses (i.e., 1.7%). Third, two variables, meaning of work and support from peers, were each assessed using a single-item measure. Future studies should consider using instruments that can measure such complex constructs because a single item might not be able to capture all aspects of the constructs [58]. Furthermore, due to the nature of the secondary data analysis, we were not able to include other potential predictors of work engagement that were not included in the KWCS dataset (e.g., work environment, size of the hospital, and years of nursing experience) in the regression models. Finally, the study data were collected using subjective measures (e.g., COPSOQ III). Future researchers may use objective measures along with self-reported measures in order to minimize the potential bias.

## Conclusion

This study demonstrated that nurse managers’ leadership and various resources were important factors for nurses’ work engagement. To promote nurses’ work engagement, nurses themselves should find meaningfulness in their work, such as fulfillment or recognition, as this helps them be more engaged in their work. Nurse managers should understand their staff’s personal values and provide feedback so that their staff can achieve their professional goals and find meaning in their nursing work. Moreover, nurse managers should use a supportive leadership style by considering individual nurses’ needs or preferences and addressing nurses’ well-being. Additionally, nurse managers should endeavor to create a supportive climate by providing positive feedback toward nurses and expressing gratitude to nurses who are supportive of other peers. Furthermore, it is essential for nursing leaders to build a fair work environment to increase nurses’ work engagement in the organizations.

## Data Availability

The data supporting the findings of this study are openly available at https://oshri.kosha.or.kr/eoshri/resources/KWCSDownload.do.

## Abbreviations

CI: confidence interval
COPSOQ III: third version of the Copenhagen psychosocial questionnaire
KWCS: Korean Working Conditions Survey
M: mean
NJD-R: Nursing Job Demands-Resources
RN: registered nurse
SD: standard deviation.
